# Impact of Injuries on Hospital Resource Utilization Among Trauma Patients Admitted due to Accidents Caused by Farm Animals

**DOI:** 10.7759/cureus.8270

**Published:** 2020-05-25

**Authors:** Rebecca Proctor, Matthew Leonard, Christy Lawson, Ha Linh, Megan Quinn, Bracken Burns

**Affiliations:** 1 Surgery, Quillen College of Medicine, East Tennessee State University, Johnson City, USA; 2 Trauma, Ballad Health Trauma Services, Johnson City, USA; 3 Surgery, Quillen College of Medicine, East Tennesse State University, Johnson City, USA; 4 Public Health, East Tennessee State University, Johnson City, USA; 5 Epidemiology and Public Health, College of Public Health, East Tennessee State University, Johnson City, USA

**Keywords:** orthopedic intervention, hospital resource utilization, farm animal injuries

## Abstract

This study examined the impact of injuries on the hospital resource utilization rate among trauma patients admitted to the Johnson City Medical Center (TN, USA), a rural Level I trauma center, due to accidents caused by farm animals. A total of 52 patients aged >3 years were studied, with the average age being 44 years. Patients above 65 years of age made up almost a quarter of the study population (23%); 63% of the patient population were male. All patients survived their injuries. Twenty-six percent of the patients required orthopedic intervention, with the majority of those patients being male (nine males, five females).

## Introduction

Farming remains one of the most dangerous occupations today and is ranked as the sixth most dangerous occupation in America [1]. Even with technological advancements, farmworkers are exposed to danger daily, particularly when working with animals. Tractor rollovers and other machinery-related injuries are more common now, but farm animal related injuries still exist. According to the US Department of Agriculture Economic Research Service, 33% of farm operators in 2012 were aged 65 or older [2]. The average age of the principal farm operator in 2012 was 58 years [2]. Males are the most common operators of farms, as women make up only 30% of the US farm operators [3]. It is unclear if resource utilization and intervention has been studied extensively, but one study highlights that out of 47 patients admitted to a single hospital following cattle-related trauma, 72% were admitted under orthopedics as their primary service provider [4]. Another study demonstrated that 59.3% of the study patients admitted required operative intervention, 78.1% of which was orthopedic surgery [5]. Orthopedics seems to be the most common care team utilized, with orthopedic surgery being the most common type of surgery required in farm-related trauma [4,5]. This study specifically attempts to further delineate hospital resource utilization from farm animal-related trauma. A rural Level I trauma center is uniquely positioned to study multisystem injury from farm animal-related trauma, which can be a big user of in-hospital resources.

## Materials and methods

This is a retrospective study that received an exempt approval from the East Tennessee State University Institutional Review Board. Electronic health data of trauma patients was obtained from the Johnson City Medical Center (TN, USA) trauma department registry. The study design was observational (cross-sectional survey). The International Classification of Diseases, Ninth Edition (ICD-9) and Tenth Edition (ICD-10) were used to obtain parameters for the study. The parameters that were studied included: • Age • Gender • Total length of stay (LOS) • Total length of stay in the Intensive Care Unit (ICU) • Total ventilator days • Injury severity score (ISS). Hospital resources were measured by length of hospital stay, total LOS in the ICU, total ventilator days, and ISS. Descriptive statistics and a regression model were used to assess the relationship between the impact of injuries and hospital resource utilization rate. The exposure variables include age, gender, LOS, and ISS. The dependent variable was the orthopedic intervention. Covariates were controlled to increase the validity of the study. The study was analysed using the IBM Statistical Package for the Social Sciences (SPSS) Statistics, version 25.0 (IBM Corp., Armonk, NY) and SAS® v9.4 (SAS Institute Inc., Cary, NC).

## Results

**Figure 1:**
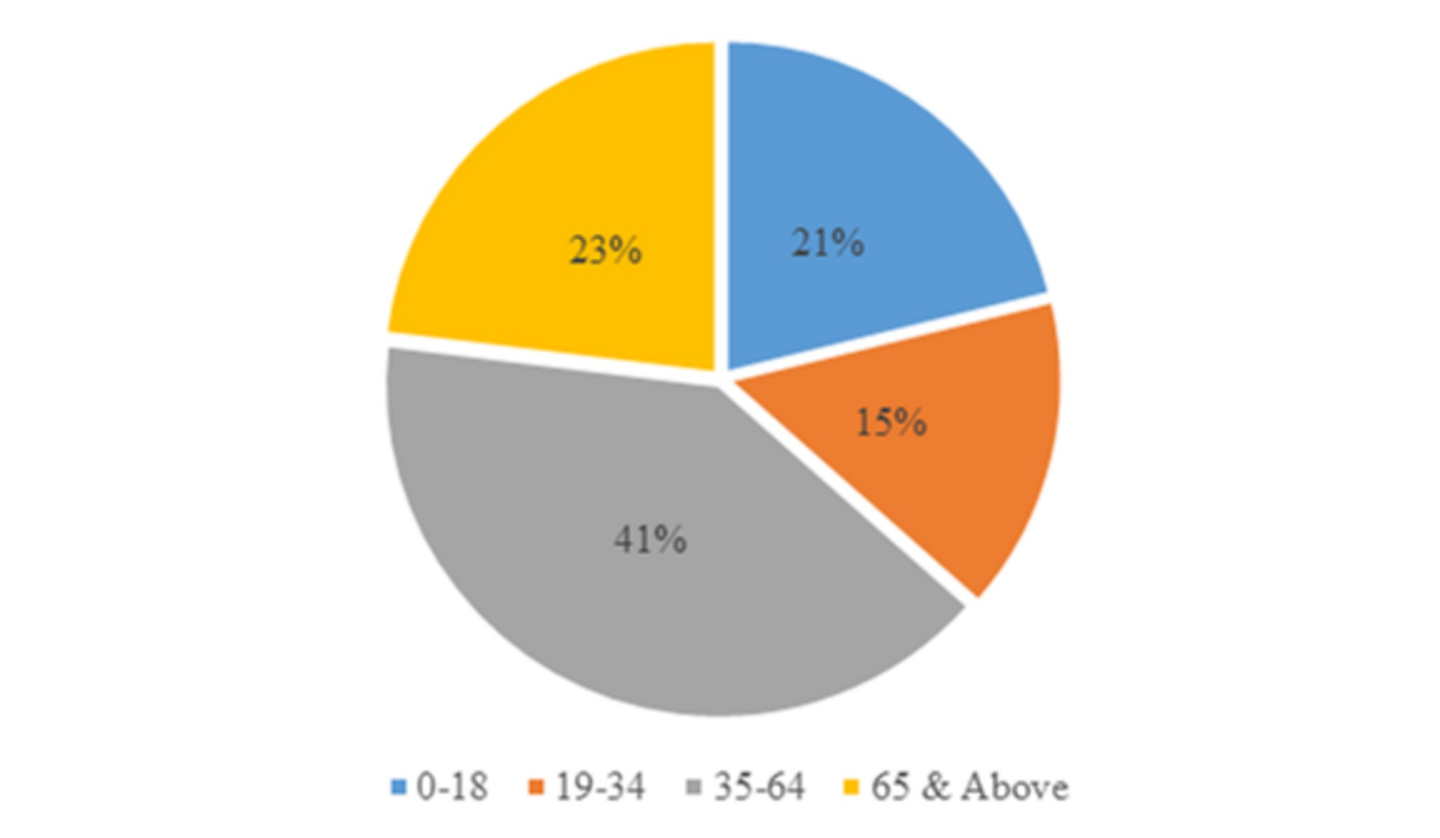
Age range distribution for farm animal injury

**Figure 2:**
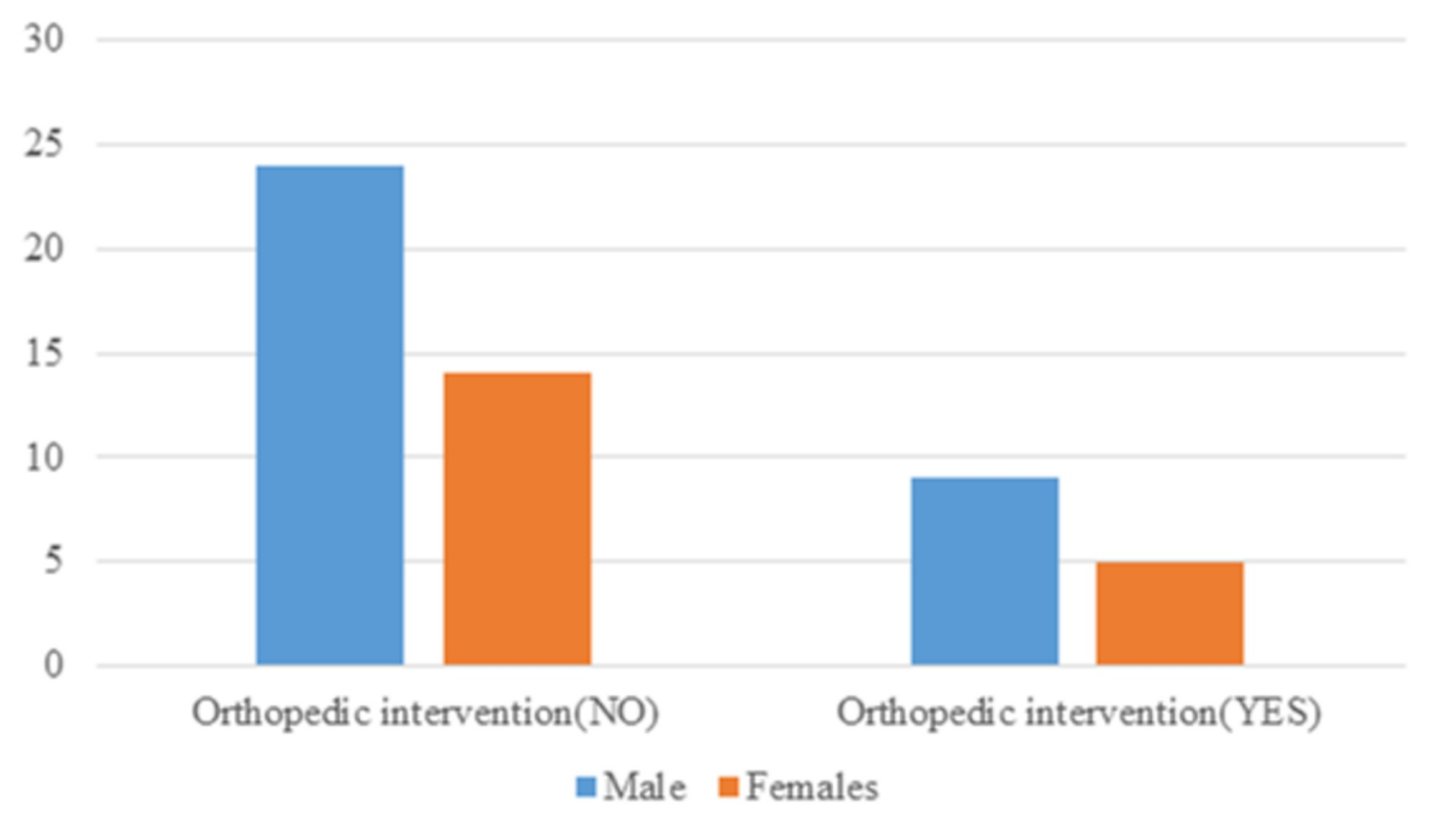
Gender distribution of patients with and without orthopedic intervention

Of the total 52 patients recruited into the study, 19 were female and 33 were male. Patients were grouped based on age. Three were within childhood age (0-9 years), eight were within adolescent age (10-18 years), eight were within young adulthood (19-34 years), 21 were within middle age (35-64 years), and 12 were within elderly age (65 years and above) (Figure 1). Of the total patients, 38 (14 female, 24 male) had no orthopedic intervention while 14 (five female, nine male) patients had some orthopedic intervention (Figure 2). All the patients survived.

The age range was 3 to 80 years, and the mean age was 44.0 years. The shortest hospital LOS was one day, while the longest was 16 days. The mean hospital LOS was 2.46 days. The shortest ICU LOS was zero days, while the longest was four days. The mean ICU LOS was 0.67 days. The shortest duration of ventilator days was zero day and the longest was three days. The mean number of ventilator days was 0.05. The lowest ISS was 1 while the highest was 21. The mean ISS was 6.68.

Out of the 52 patients, 13 patients (8 males, 5 females) stayed for more than the average length of hospital stay of 2.46 days, while 39 patients (25 males, 14 females) stayed less than the average hospital length of stay. Eighteen of the 52 patients (11 males, 7 females) had an ISS above the average score of 6.68, while 34 (23 males, 11 females) had an ISS below average. Out of the nine patients with an ICU stay, all (four males, five females) had an ICU LOS above the average score of 0.67 days (Table 1).

**Table 1:** Mean distribution of study parameters for the impact of injuries due to farm animals LOS, length of stay; ICU, intensive care unit; ISS, injury severity score

Variables	Observations (N)	Min	Max	Mean	SD	Percentage
Age	52	3	80	44.0	43.73	-
Total LOS	52	1	16	2.67	3.34	-
Total ICU days	9	0	3	0.67	0.91	-
ISS	52	1	21	6.39	4.95	-
Orthopedic intervention (no)	38	-	-	-	-	73
Orthopedic intervention (yes)	14	-	-	-	-	27
Discharge status (alive)	52	-	-	-	-	100

A logistic regression analysis was run between the categories of age, gender, LOS, ISS, and orthopedic interventions to determine whether there was a significant difference between the specified populations. The odds of orthopedic interventions were 36.4% more likely for females when compared to males, while other variables in the model were constant (odds ratio [OR] = 1.364, 95% CI = 0.32, 5.85). The odds of orthopedic intervention were 0.5% more likely with every one-unit increase in age, while other variables in the model were constant (OR = 1.005, 95% CI = 0.97, 1.04). The odds of orthopedic intervention were 64% less likely with every one-unit increase in the length of hospital stay, while other variables in the model were constant (OR = 0.36, 95% CI = 0.15, 0.84). The odds of orthopedic intervention were 3.31 times more likely with every one-unit increase in the length of ICU stay, while other variables in the model were constant (OR = 3.31, 95% CI = 0.61, 17.78). The odds of orthopedic intervention were 72% more likely with every one-unit increase in the length of ventilator days, while other variables in the model were constant (OR = 0.284, 95% CI = <0.001, >999.999). The odds of orthopedic intervention were 3.6% more likely with every one-unit increase in ISS, while other variables in the model were constant (OR = 0.964, 95% CI = 0.776, 1.20).

## Discussion

Overall, there is a relationship between age and hospital resource utilization rate, with increasing age correlating with increased utilization rates. The descriptive statistics showed that most of our study population falls within the middle-age group and predominantly male gender. Males represented the majority of injuries across multiple studies observed [4-7]. However, despite our study population being predominately male, females were more likely to have an orthopedic intervention in this study. Each category showed a positive unit increase in the need for orthopedic surgery except for the length of hospital stay. Unfortunately, the sample size for this study is small, which is a limitation of this study design. If a larger sample size were used, there might have been some significant differences between the utilization of hospital resources and length of hospital stay. Also, unequal gender distribution may be responsible for the mild gender differences in hospital resource utilization noted in this study group.

There is limited research on hospital resource utilization in farm animal-related trauma. The results of this study varied slightly compared to the available literature. It seems more common for farm animal-related injuries to occur in middle-age patients between 35 and 65 years of age, as demonstrated in our study and two others [4,5]. Casey et al. in a study noted that of their 57 patients, 46 were aged between 23 and 58 years [6]. The population that was evaluated in this study required significantly less orthopedic interventions than noted in other study groups. Age could be a factor in the decreased orthopedic interventions, as the mean age in this study group was 44 years. Comparing our results to other published studies, we showed a mean age of 44 years compared to mean ages of 56 and 53 years. We also showed less operative interventions at 26% compared to 59% and 78% in other published studies [4,5]. Length of ICU days could also be a factor, as this study reported that the mean LOS was only 0.67 days compared to 4 and 12.3 days in other studies [4,5]. Mortality risk remains low among farm animal-related injuries in this study group, which is supported by the literature [5].

## Conclusions

In this study, farm animal-related trauma more commonly affected those aged 35-64 years. Orthopedic intervention is the most common type of hospital resource required and is more likely to be utilized in females than males. With an increase in age or ICU days, the need for orthopedic interventions also increases. There was no statistically significant increase in the need for intervention based on ISS or hospital LOS. There is a paucity of literature regarding hospital resource utilization in farm animal-related trauma, and further studies are warranted. A larger sample size could greatly benefit future studies.
